# Adaptability assessment of *Aspergillus niger* and *Aspergillus terreus* isolated from long-term municipal/industrial effluent-irrigated soils to cadmium stress

**DOI:** 10.1186/s12866-025-04000-9

**Published:** 2025-05-15

**Authors:** A. Metwally Rabab, S.Taha Asmaa, H. Mohamed Asmaa, A. Soliman Shereen

**Affiliations:** Botany and Microbiology Department, Faculty of Science, Zagazig University, Zagazig, 44519 Egypt

**Keywords:** *Aspergillus niger*, *A. terreus*, Antioxidant enzymes, Heavy metals, Tolerance index, Bioaccumulation capacity

## Abstract

Heavy metals (HMs) contamination is a major issue produced by industrial and mining processes, among other human activities. The capacity of fungi to eliminate HMs from the environment has drawn attention. However, the main process by which fungi protect the environment against the damaging effects of these HMs, such as cadmium (Cd), is still unknown. In this study, some fungi were isolated from HMs-polluted soil. The minimum inhibitory concentrations (MICs) and the tolerance indices of the tested isolates against Cd were evaluated. Moreover, molecular identification of the most tolerant fungal isolates (*Aspergillus niger* and *A. terreus*) was done and deposited in the GenBank NCBI database. The results showed that the colony diameter of *A. niger* and *A. terreus* was decreased gradually by the increase of Cd concentration. Also, all the tested parameters were influenced by Cd concentration. Lipid peroxidation (MDA content) was progressively increased by 12.95–105.95% (*A. niger*) and 17.27–85.38% (*A. terreus*), respectively, from 50 to 200 mg/L. PPO, APX, and POD enzymes were elevated in the presence of Cd, thus illustrating the appearance of an oxidative stress action. Compared to the non-stressed *A. niger*, the POD and PPO activities were enhanced by 92.00 and 104.24% at 200 mg/L Cd. Also, APX activity was increased by 58.12% at 200 mg/L. Removal efficiency and microbial accumulation capacities of *A. niger* and *A. terreus* have also been assessed. Production of succinic and malic acids by *A. niger* and *A. terreus* was increased in response to 200 mg/L Cd, in contrast to their controls (Cd-free), as revealed by HPLC analysis. These findings helped us to suggest *A. niger* and *A. terreus* as the potential mycoremediation microbes that alleviate Cd contamination. We can learn more about these fungal isolates’ resistance mechanisms against different HMs through further studies.

## Introduction

Industry and urbanization are to blame for environmental pollution in the soil, air, and water [1, 2]. These operations produce enormous amounts of heavy metal (HM) ions, which can cause several health problems in humans, animals, and other living organisms [3–5]. HMs are distinguished by having a higher atomic weight or density of over 5 g/cm^3^ and can be classified into essential HMs such as zinc (Zn), iron (Fe), copper (Cu), and manganese (Mn), and non-essential HMs such as arsenic (As), cadmium (Cd), chromium (Cr), lead (Pb), and nickel (Ni) [6, 7].

One of the commonly distributed metals is Cd, which is biologically non-essential and hazardous to humans, plants, microbes, and animals [8, 9]. For a wide variety of plants, Cd is poisonous. According to Shan et al. [10] and Liu et al. [11], the long-term effects of Cd may substantially impair agricultural crop output and quality in addition to invading human bodies through the food chain; it can continue to be hazardous for over 20 years [12]. It has been demonstrated that Cd affects photosynthesis, transpiration, and stomatal opening in plants cultivated in nutrient solutions [13]. Moreover, long-term Cd exposure damages the lungs and causes renal tubular failure in humans. In addition, Cd poisoning has a major impact on cardiac health and causes significant biochemical and physiological alterations [14].

However, Limcharoensuk et al. [15] reported that certain soil microbes have evolved defense mechanisms in highly Cd-polluted environments; furthermore, they may reduce their bioavailability. As a result of increased usage of fertilizers and pesticides in modern agriculture and industry, the amount of Cd released into the environment has increased, making it easy for plants to accumulate it because of its high mobility [4, 16]. The HMs are extremely poisonous, non-biodegradable, and persistent in the environment; getting rid of them is critical. In comparison to other currently employed conventional technologies such as adsorption onto activated carbon and metal oxides, chemical precipitation, membrane filtration, and ion exchange, the bioremediation process has demonstrated numerous advantages that make it a relatively cost-efficient, effective approach and an environmentally sustainable method that uses fewer natural resources [5, 17–19].

Bioremediation is a long-term strategy for treating polluted environments depending on the metabolic potential of microorganisms, where HMs can be adsorbed and accumulated by all microorganisms, such as bacteria, fungi, and algae [20–23], due to the ability of their cells to deal with large levels of HMs, active absorption, and accumulation [5, 24]. They convert toxic HMs into a less damaging form utilizing microbes or their enzymes [25, 26]. One of the most promising microbes in bioremediation technology is the use of fungi, as they are characterized by large cell size, high surface-to-volume ratio, fast growth, high biomass production, remarkable metal tolerance capacity, high metal-binding efficiency within the cell and on the surface of the cell wall, and the production of extracellular enzymes to counter metal ions [5, 23, 27, 28]. Fungi also are excellent candidates for metal uptake since their cell walls contain glucan, chitin, proteins, and lipids with significant functional groups (carboxylate, hydroxyl, sulphate, phosphate, and amino) [28, 29]. Metal tolerance in filamentous fungi has been related to their isolation sites, metal toxicity, and metal concentration in the medium, as well as the isolate’s competence [30].

Fungi have evolved extracellular and intracellular mechanisms for maintaining homeostasis to virtually all toxic HMs [22, 26]. Extracellular mechanisms incorporate avoiding metal entry into the cell [31]. On the other hand, metal detoxification and sequestration are part of the intracellular mechanism, which is mediated by the interaction of metals with cytosolic peptides, like reduced glutathione (GSHs), Phytochelatins (PCs), and metallothioneins (MTs) [26, 32], and polyphosphate granules or retain it in the vacuoles [33]. The synthesis of enzymatic antioxidants such as peroxidase (POD), catalase (CAT), and polyphenol oxidase (PPO) [34, 35] operates as reactive oxygen species (ROS) scavengers. Moreover, non-enzymatic antioxidants such as proline and total soluble carbohydrates are the additional defensive systems that lessen oxidative stress generated by ROS by eliminating their intermediates and blocking further oxidation events [31, 36]. Owing to its great tolerance and resilience to various metal ions, the *Aspergillus* genus is utilized in microbial remediation to remove HMs [37–39]. This work was carried out to isolate fungi from HM-polluted soils from the El-Sharkia governorate of Egypt and evaluate the resistance level of the most tolerant isolates toward Cd, in addition to investigating their microbial accumulation efficiency to gain information about bioremediation mechanisms and the Cd toxicity influence on the physiology of the most tolerant isolates.

## Materials and methods

### Soil samples collection and HMs analysis

To assess the tolerance potential of fungal isolates against Cd, soil samples were collected separately in sterile plastic bags from Meet-Gaber village in El-Sharkia Governorate, which received a long-term application of untreated municipal/industrial effluents. Soil samples were collected at a depth of 25 cm. After that, soil samples were maintained at 4^o^C and further used to isolate fungi.

Moreover, for HM analysis, 1 g of the soil was transferred to a digestion tube and then digested with 10 mL of HCl: HNO_3_, a 3:1 v/v mixture. The flasks were heated until the clarity of the digest. After filtration, the extracts were brought to a volume of 25 mL by deionized water. The concentration of Cd, Ni, Mn, Zn, and Pb in the soil was evaluated with an atomic absorption spectrophotometer of the Faculty of Veterinary Medicine, Zagazig University, Zagazig City.

## Fungal isolates, and its Cd- tolerance and identification

### Fungal isolation

The serial dilution technique was used to isolate fungal isolates from HM-contaminated soils irrigated with untreated municipal/industrial effluents. 10 g of soil samples were mixed with 90 mL of sterilized water and diluted up to 10^− 6^. From each dilution, 0.1 mL was spread on potato dextrose agar (PDA) media and incubated at 28 ^o^C. To limit the bacterial growth, streptomycin (15 mg L^− 1^), and chloramphenicol (50 mg L^− 1^) were added after sterilization. Regularly, Petri plates were observed, and fungal mycelia were subcultured on fresh PDA. The purified fungal samples were stored at 4 ^o^C.

### Fungal screening for Cd- tolerance

The Cd tolerance of fungal strains isolated from contaminated soils was determined by adding 100 mg L^ˉ1^ CdCl_2_ to the PDA medium. The medium was adjusted to pH 6 with 1 M NaOH solution. Agar discs (8 mm) from the active growing edge of the fungal cultures were cut and inoculated to the surface of PDA plates. Meanwhile, PDA plates without Cd were performed as a control. All the plates were incubated at 28 °C for 7 days. The radial growth was evaluated in mm. The growth rate (kd) of fungal isolates was calculated by the following equation: kd = D/T, where D is the average diameter of the fungal colony in mm and T: time in hours (h). Moreover, the tolerance index (TI) of fungal isolates, as an indication of the fungal response to metal, was measured from the diameter of the colony in the presence of metal divided by the diameter of the colony in the control plate [40]. Fungal tolerance was rated as: 0.00 to 0.39 (very low tolerance), 0.40 to 0.59 (low tolerance), 0.60 to 0.79 (moderate tolerance), 0.80 to 0.99 (high tolerance), and 1.00 to > 1.00 (very high tolerance) [41]. The tolerance index was calculated according to the following equation:

$$\eqalign{& {\rm{Tolerance\: index }}({\rm{TI}}) \cr& = {{{\rm{ Diameter\: of\: fungal\: colony\: in\: the\: presence\: of\: metal\: }}} \over {{\rm{ Diameter\: of\: fungal\: colony\: without\: metal\: exposure }}}} \cr& \times 100 \cr} $$

### Identification of fungal isolates

The fungal isolates that showed a high Cd tolerance were identified based on morphological and molecular techniques. For the microscopic identification, a light microscope (Leitz WETZLAR, Germany) was used.

The pure cultures of fungal isolates were grown on a PDA medium for 7 days at 25 °C for molecular identification. DNA was extracted using the CTAB technique [42]. The ITS1, ITS2, and the interspaced 5.8 S were amplified according to White et al. [43] using the ITS1 and ITS4 primers. Before DNA sequencing, the PCR amplicon was examined using 0.8% agarose gel and purified using a PCR purification kit (Accu Prep^®^ PCR DNA Purification Kit, K-3034-1, Bioneer Corporation, South Korea). Macrogen Inc, (South Korea) sequenced the purified PCR products using an ABI PRISM ^®^ 377 DNA auto sequencer (PerkinElmer, Applied Biosystems Div., Waltham, USA).

## Tolerance studies of most potent fungal strains

To explore the response of the most potent strains to Cd stress, TI, dry weight, removal efficiency, bioaccumulation capacity, lipid peroxidation, and enzymatic and non-enzymatic antioxidants were determined.

### Fungal growth and minimum inhibitory concentration (MIC) of Cd- tolerant strains

To determine radial growth and MIC of Cd-tolerant strains that inhibited visible growth, different Cd concentrations were used according to Xu et al. [44]. PDA medium was supplemented with different concentrations of Cd (0, 50, 100, 150, 200, 250, 300, 350, 400, and 450 mg L^ˉ1^ Cd). The plates were seeded with agar plugs from a 7-day-old pure fungal culture. If no growth of fungi was noticed after 10 days, the metal concentration was regarded as the highest metal concentration tolerated by the tested fungus. Moreover, the radial growth was determined in mm.

### Cd bioaccumulation and removal efficiency from liquid media by tolerant fungi

The growth pattern, removal efficiency (Re%), and bioaccumulation (metal uptake) capacity (Q) of the fungal isolates were investigated in liquid cultures. Erlenmeyer flasks of 250 mL containing 100 mL of sterilized potato dextrose broth (PDB) at pH 5.6 ± 0.2, with various concentrations of Cd ranging from 50 to 200 mg Cd(II) L^− 1^ were separately added. Flasks were inoculated with 8 mm disks from 7-day-old pure fungal culture. Untreated flasks (PDB medium without Cd) were served as controls. All flasks were incubated at 28 °C at 120 rpm. After 10 days of incubation, according to Gruhn and Miller [45], fungal biomass was harvested and filtered through Whatman No. 1. The residual Cd concentration in the filtrate was used for estimating Cd concentration using an atomic absorption spectrophotometer (AAS) (Unicam 969, Central Laboratory, Faculty of Veterinary, Zagazig University, Zagazig City). Fungal biomass was rinsed 3 times with distilled water and dried at 80 °C until a constant weight. The fungal biomass was then weighed and defined as dry biomass (g). The Re% and Q were calculated according to Pan et al. [46] and Javaid et al. [47] by the following equations:

$$\operatorname{Re}(\%)=\frac{\mathbf{C i}-\mathbf{C f}}{\mathbf{C i}} \times 100$$

$$Q=\frac{C i-C f}{m} X V$$

Re: removal efficiency; Q: bioaccumulation (metal uptake) capacity.

Where Ci and Cf are the initial and final concentrations of Cd (mg L^− 1^), Q is the metal uptake (mg/g dry weight of fungal biomass), and Re is the Cd removal rate; m is the dry weight (g) of fungal biomass, and V (L) is the initial volume of aqueous medium.

### Stress marker (Malondialdehyde [MDA] content)

The MDA content as a lipid peroxidation product was measured in 0.25 g of the fresh fungal mycelia according to Heath and Packer [48] after being homogenized in 5 mL of 0.1% trichloroacetic acid (TCA) and centrifuged at 6000 rpm for 15 min. Subsequently, 2 mL of supernatant was added to 4 mL of 0.5% thiobarbituric acid (TBA) in 20% TCA at 95 °C for 30 min. The reaction was cooled immediately on ice and centrifuged; it formed a pinkish-red pigment comprising two molecules of TBA and MDA that measured photometrically at 532 and 600 nm. The MDA content was calculated by the following equation:

$$\text { MDA } \cdot \text { content }=\cdot\left(\mathrm{A}_{532}-\mathrm{A}_{600} / \mathbf{1 5 5}\right)^* 1000$$

### Enzymatic antioxidants

For the protein and anti-oxidative studies, fungal biomass (1 g of fresh weight [fwt]) was crushed with 10 mL of 0.1 M potassium phosphate buffer (PPB) (pH: 7.0) containing 50 mM EDTA in an ice-cold mortar. The homogenate was centrifuged at 6000 rpm for 15 min. The supernatant was collected for total soluble protein and antioxidant enzyme assay.

Ascorbate peroxidase activity (APX) was analyzed using the Nakano and Asada [49] technique; 250 µL of fungal supernatant was added to a 3 mL reaction mixture consisting of 2.5 mL of 0.1 M PPB (pH 7.0), 0.1 mL L-ascorbate, and 0.15 mL H_2_O_2_. The absorbance was recorded at 290 nm spectrophotometrically against a blank and its activity was calculated using the molar extinction coefficient of ascorbate (absorbance of one molar solution) (2.8 mM^− 1^ cm^− 1^).

Peroxidase (POD) activity was analyzed at 470 nm by Chance and Maehly [50]. The reactant mixture contained 0.1 M PPB (pH 7.0), 1% guaiacol, 0.4% H_2_O_2,_ and 250 µL fungal supernatant. According to Beyer and Fridovich [51], the polyphenol oxidase (PPO) activity in 250 µL enzyme extract of both fungi under different Cd concentrations was subjected to the reaction with 0.1 M PPB (pH 7.0) and 100 µM pyrogallol. The absorbance was measured at 430 nm, and its activity was expressed as U g^− 1^ fwt.

### Non-enzymatic antioxidants [proline, protein, and total soluble carbohydrates (TSC) contents

The proline content in 0.25 g fwt of fungal biomass was determined [52] at 520 nm after homogenizing it in 3% aqueous sulphosalicylic acid. Two mL of the fungal filtrate was mixed with 2 mL of acid ninhydrin and 2 mL of glacial acetic acid in a boiling water bath for 60 min. To stop the reaction, the mixture was left to sit in the ice bath. After adding 4 mL of toluene to the mixture, the absorbance was measured, and proline content was given as µM g^− 1^ fwt. The method of Lowry et al. [53] was adopted in 1 mL of fungal extract after mixing with 1 mL of freshly prepared alkaline copper solution and incubated for 15 min at room temperature to determine the total soluble proteins using Folin’s reagent. The absorbance of the developed blue color was measured at A650 nm, and its concentration was expressed as mg/g fwt. In addition, the TSC level was estimated using the Dubois et al. [54] technique. After combining 100 mg of dry mycelial mats with 2.5 N HCl for three hours in a boiling water bath, the mixture was centrifuged for ten minutes at 5000 rpm. Next, 1 mL of the supernatant was mixed with 5 mL of H_2_SO_4_ and 1 mL of 5% phenol. After vigorous agitation and 30 min of cooling, the absorbance was read at 485 nm.

### Assay of total thiol, non-protein thiol and protein thiol

The amounts of total thiols, non-protein thiol, and protein thiol in 1 g of fresh fungal mycelia were assessed by Sedlak and Lindsay’s [55] procedure using Ellman’s Reagent after homogenizing in 10 mL of 0.2 M Tris-HCl (pH 7.4) and centrifuging at 8000 rpm for 15 min at 4 °C. The fungal extracts (0.5 mL) were mixed well with 1.5 mL of 0.2 mM Tris-HCl (pH 8.2), 0.1 mL of 0.01 M DTNB (Ellman’s Reagent) (5, 5-dithio-bis-(2-nitrobenzoic acid), and 7.9 mL of absolute methanol to develop yellow color which was measured after 15 min at 415 nm to determine the total thiol. Total sulfhydryl groups were expressed as mg/g fwt using an extinction coefficient of 13,600. 5 mL of the supernatant was mixed with 4 mL of distilled water and 1 mL of 50% TCA to evaluate the non-protein thiol content. After 15 min, the mixture was centrifuged at 8000 rpm for 15 min. Non-protein thiol concentration was determined in 2 mL of deproteinized supernatant in the same way as total thiol. The protein thiol content was calculated by subtracting the non-protein thiol content from the total thiol content.

### Organic acids by high performance liquid chromatography (HPLC)

To determine the role of organic acids in Cd tolerance, oxalic, tartaric, succinic, malic, and citric acids were detected in the culture filtrates grown in Cd-free and Cd-stressed conditions (200 mg/L). An Agilent chromatograph, equipped with SUPELCOGEL C-610 H, 30 cm x 7.8 mm ID, was used for the analysis, with data collected by a ChemStation. The mobile phase was 0.1% H_3_PO_4_ with a flow rate of 0.5 mL/min. The organic acids were evaluated by comparing the absorbance at 210 nm to the standard concentrations of the three organic acids.

### Data analysis

The statistical analysis was performed using SPSS software (Version 16.0, SPSS Inc., Chicago, IL, USA). Values shown in this work are the means ± standard deviation. All treatments were carried out with a triplicate sample. The difference in TI and uptake capacity of each isolate was studied by one-way ANOVA followed by post-hoc multiple comparisons by Duncan’s method. The difference was considered significant when *p* < 0.05.

## Results and discussion

### HMs analysis in soil

Human activity is the primary cause of pollution, and it results in HMs pollution. It is mostly caused by mining metal, foundries, and other metal-based industries as well as the leaching of metals from sites like landfills [56]. The HM concentration in soil from the study area has been assessed. The obtained data are presented in Table 1, which demonstrates that the soil is positive for HMs. Table (1) shows the concentration of Cd, Pb, Ni, Zn, and Cu in the contaminated soils where their values exceeded the maximum permissible concentration for HMs [57] with concentrations: 25.8, 135.2, 57.2, 284.2, and 94.5 mg/kg, respectively. Generally, the results of the HMs concentration revealed that the soil is contaminated with a high concentration of Cd, Cu, Pb, and Zn.

**Table 1:** HMs concentration in soil sample

	Total content of HMs (mg/kg)				
	Pb	Cd	Ni	Zn	Cu
Soil sample	135.2	25.8	57.2	284.2	94.5
The maximum permissible concentration for HM in soil WHO (1996)	85	0.8	35	50	36

### Cd tolerance

**Fig. 1:**
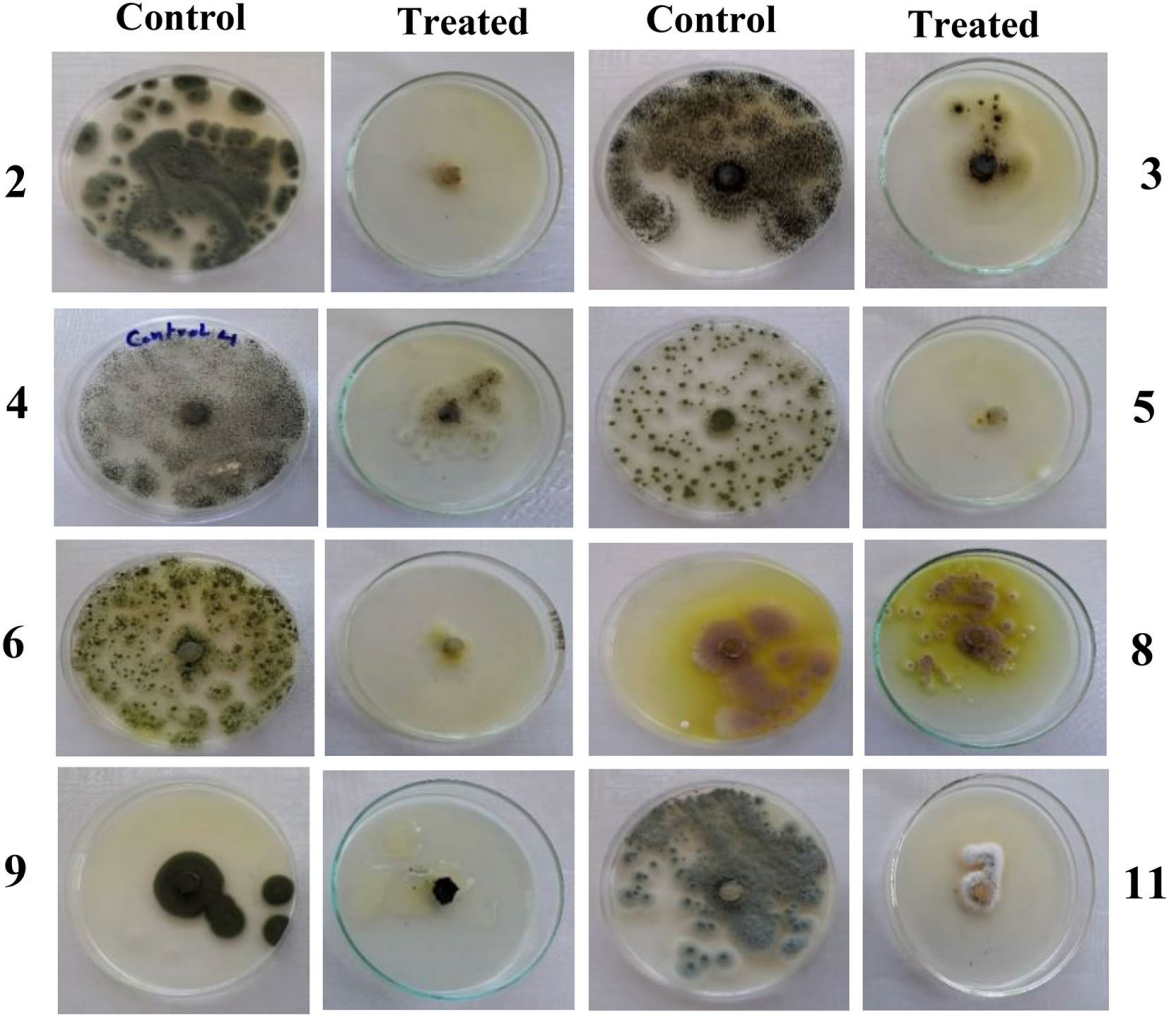
The preliminary qualitative screening of fungal isolates on Cd (100 mg/L)- supplemented agar plates. Notice that isolates number 3 and 8 were the most tolerant to 100 mg/L CdCl_2_ as compared to the others

**Fig. 2:**
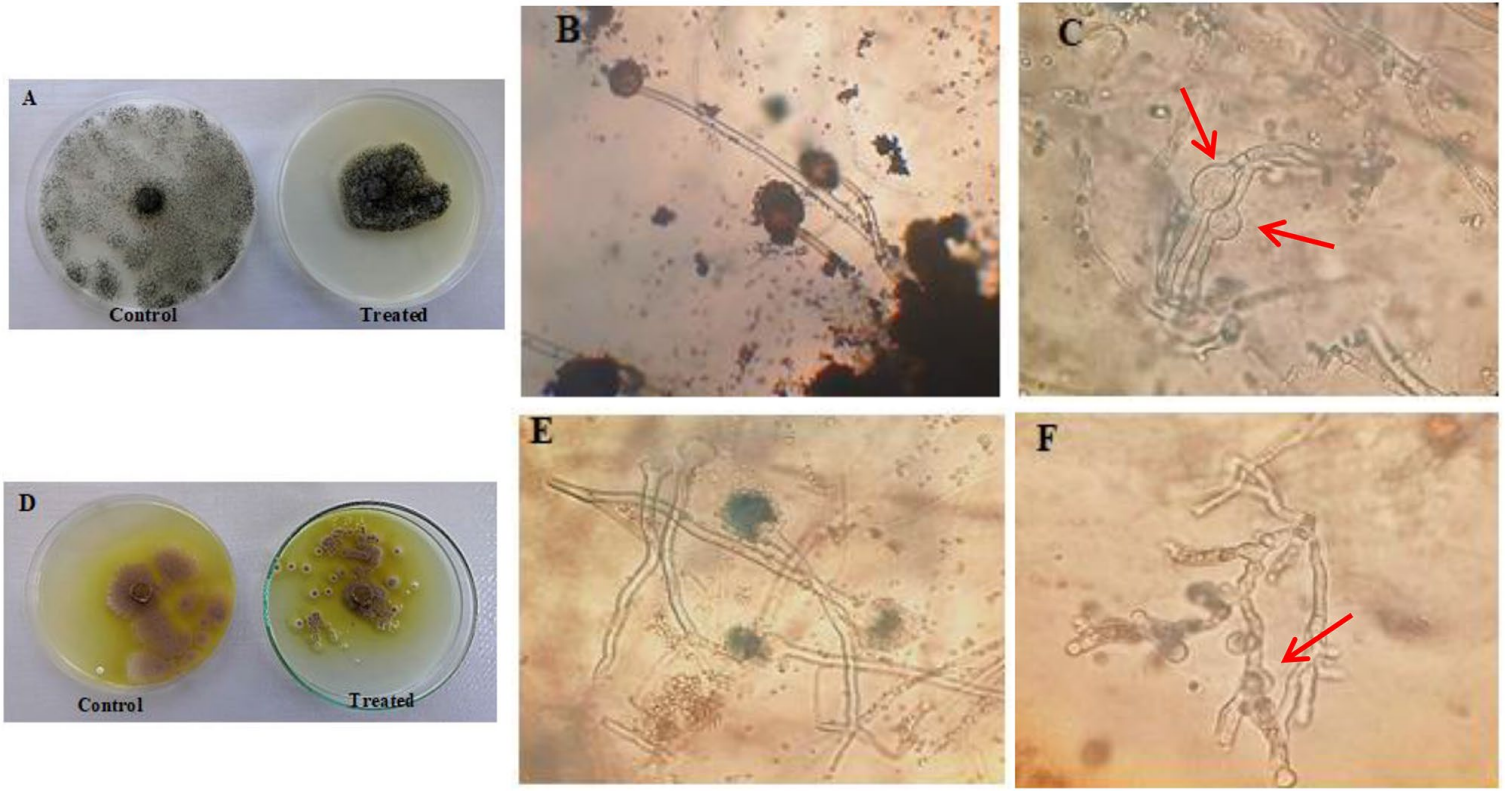
(**A**) and (**D**) show the growth of *A. niger* and *A. terreus*, respectively on PDA media supplemented with CdCl_2_ (100 mg/L), (**B**) and (**E**) show the morphological normal untreated hypha (Control) of *A. niger* and *A. terreus*, (**C**) and **(F)** show morphological abnormalities in the fungal mycelia as intensively swelling deformed mycelium of *A. niger* and segmented deformed mycelium of *A. terreus* under Cd stress. Picture was taken after 7 days of incubation at 28 °C

**Fig. 3:**
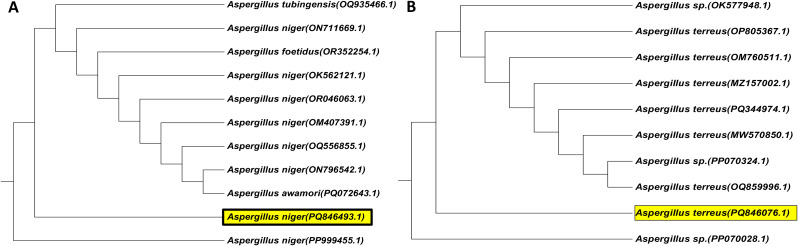
The phylogenetic tree of the 18 S rRNA genes for *A. niger* (**A**) and *A. terreus* (**B**) and the others presented on GenBank based on the DNA sequence

The screening of Cd resistance was performed with twelve fungal isolates isolated from Meet-Gaber village contaminated soil that received a long-term application of untreated municipal/industrial effluents. The selectivity of the isolates was determined by their capacity to develop on a PDA medium supplemented with 100 mg/L CdCl_2_. Table (2) shows the response of 12 fungal isolates against Cd as colony diameter (mm) and sporulation (high, medium, or low). The preliminary qualitative screening results (Table 2) declared that 11 isolates showed visible growth on Cd plates. The sporulation was represented as high, medium, and low in response to Cd. Also, the growth of fungal isolates was represented as negative results (-) and positive results (+) with varying degrees as very good growth (+++), good growth (++), and visible growth (+). Among these isolates, isolates assigned with numbers 3, 4, 8, and 12 showed higher colony diameter (growth) for Cd in comparison to the rest of the isolates (Tables 2 and 3) in the following order: 3 > 8 > 4 > 12 (Fig. 1). The highest colony diameter was recorded for isolate nos. 3 and 8, which were identified morphologically as *Aspergillus niger* and *A. terreus* according to Raper and Fennell [58] (Fig. 2). For molecular identification, the pure culture of the most potent fungal isolates (*A. niger* and *A. terreus*) that showed a high Cd tolerance was identified using the 18 S rDNA gene sequence. The obtained partial sequence of the 18 S rDNA gene was deposited in the GenBank database under accession numbers PQ846493 and PQ846076, respectively, as shown in Fig. 3.

**Table 2:** Qualitative screening of fungal isolates for cd resistance

Fungal isolates	Growths	Sporulation
1	-	-
2	+	Low
3	+++	High
4	++	Medium
5	+	Low
6	+	Low
7	+	Low
8	+++	High
9	+	Low
10	+	Low
11	+	Low
12	++	Medium

-: no visible growth, +: visible growth, ++: good visible growth and +++: very good visible growth

**Table 3:** Preliminary responses of fungal isolates to 100 mg/l CdCl_2_, plates were incubated at 28 °C for 7 days

Fungal isolates	Absence of CdCl_2_ ( control )	Presence of 100 mg/L CdCl_2_	TI
	Colony area diameter (cm)	Colony diameter (cm)	
1	5.46 ± 0.144b	0 h	0
2	8.20 ± 0.216a	0.53 ± 0.014e	6.46
3	8.20 ± 0.216a	5.03 ± 0.133a	61.34
4	8.20 ± 0.216a	3.97 ± 0.105c	48.41
5	8.20 ± 0.216a	0.43 ± 0.011ef	5.24
6	8.20 ± 0.216a	1.80 ± 0.048d	21.95
7	3.96 ± 0.104c	0.46 ± 0.012ef	11.61
8	5.33 ± 0.141b	4.53 ± 0.120b	84.99
9	2.13 ± 0.056d	0.27 ± 0.007 g	12.67
10	0.96 ± 0.025e	0.53 ± 0.014e	55.20
11	8.20 ± 0.216a	1.90 ± 0.050d	23.17
12	8.20 ± 0.216a	3.97 ± 0.105c	48.41

*The data in the table was mean ± standard error of the means (*N* = 3). Letters indicate statistical significance at *p* ≤ 0.05 level

Fungal isolates displayed growth inhibition that may be related to metal toxicity, and the reduction in fungal development may be caused by an increase in the lag phase period compared to the control [59]. Our results of screening confirm those of Dwivedi et al. [60] and Abd El Hameed et al. [61]. Rose and Devi [17] and Ezzouhri et al. [62] screened HM-resistant fungi and noticed that the most tolerant fungi belonged to the genus *Aspergillus*, as it is an efficient reducer of HMs. Moreover, Iram et al. [59] isolated fungi from industrial effluent-irrigated agricultural soil and identified 13 isolates as *A. niger*. The simultaneous appearances of different *Aspergillus* species may be due to their being adapted to their environment, producing a significant amount of easily dispersed spores. The ability to grow on such high concentrations of toxic metals indicates that the obtained fungal isolates are considered HM-tolerant fungi [63]. From screening results, *A. niger* and *A. terreus* showed superior growth on Cd-supplemented media (100 mg/L). Therefore, these two fungal isolates were molecularly identified and selected for subsequent studies.

### Effects of cd different concentrations on the growth profile and MIC of *A. niger* and *A. terreus*

Firstly, the behavior of both fungal isolates was evaluated on the growth characteristics by increasing the Cd concentration in the medium. The assessment of a microorganism’s resistance is typically estimated by measuring the MIC as the lowest metal concentration that stops the fungi from growing after culturing on both liquid and solid growth media [9]. The MIC and the radial growth of the most potent fungal isolates on PDA with different Cd concentrations are shown in Table 4. Generally, the colony diameter of *A. niger* and *A. terreus* was decreased gradually by the increase of Cd concentration. *A. niger* and *A. terreus* continued their growth till the concentration of 450 and 350 mg/L, which represented MIC for *A. niger* and *A. terreus*, respectively (Table 4). Thus the Cd tolerance for *A. niger* was greater than that of *A. terreus* (Table 5). The values of MIC recommend that the level of tolerance against Cd was reliant on the type of isolates, as the two species of *Aspergillus* exhibited a noticeable difference in the level of Cd tolerance that may be related to distinct tolerance mechanisms shown by diverse fungi [17]. Moreover, the growth of *A. niger* and *A. terreus* on PDB did not follow the same behavior on PDA, which might be due to the formation of HMs gradients in agar that give a protective chelating effect [28, 64]. The biomass of *A. niger* and *A. terreus* was slightly decreased (2 and 3.28%, respectively) at 50 mg/L Cd, while at a concentration of 200 mg/L Cd, the fungal growth was progressively diminished (15.12 and 27.87%, respectively), while no growth was detected at 250 mg/L Cd (Table 6). Growth suppression may come from the increased cadmium stress, which may disrupt protein synthesis and enzyme activity as well as trigger autophagy and apoptosis [65].

**Table 4:** Effect of different CdCl_2_ Conc. On the colony area and growth rate (kd) of *A. niger* and *A. terreus* On PDA medium

Cd conc. (mg/kg)	*A. niger*				*A. terreus*			
	Colony area (cm) 5 days	Growth rate (kd)	Colony area (cm) 10 days	Growth rate (kd)	Colony area (cm) 5 days	Growth rate (kd)	Colony area (cm) 10 days	Growth rate (kd)
0	8.20 ± 0.216a	1.64	8.20 ± 0.216a	0.82	7.03 ± 0.186a	1.40	8.20 ± 0.216a	0.82
50	4.70 ± 0.124b	0.94	8.20 ± 0.216a	0.82	2.56 ± 0.067b	0.51	8.20 ± 0.216a	0.82
100	3.86 ± 0.102c	0.77	8.20 ± 0.216a	0.82	0.43 ± 0.011c	0.08	0.43 ± 0.011d	0.04
150	3.70 ± 0.097c	0.74	3.70 ± 0.097b	0.37	0.40 ± 0.010c	0.08	0.40 ± 0.010d	0.04
200	3.06 ± 0.081d	0.72	3.06 ± 0.081c	0.30	0.40 ± 0.010c	0.08	1.56 ± 0.041b	0.15
250	2.26 ± 0.059e	0.45	2.26 ± 0.059d	0.22	0.16 ± 0.004d	0.03	0.16 ± 0.004de	0.01
300	0.90 ± 0.023f	0.19	0.90 ± 0.023e	0.09	0.56 ± 0.014c	0.11	1.03 ± 0.027c	0.01
350	0.60 ± 0.015 g	0.12	0.60 ± 0.015f	0.06	0d	0	0e	0
400	0.50 ± 0.013 g	0.1	0.50 ± 0.013f	0.05	0d	0	0e	0
450	0 h	0	0 g	0	0d	0	0e	0

*The data in the table was mean ± standard error of the means (*N* = 3). Letters indicate statistical significance at *p* ≤ 0.05 level

**Table 5:** Effect of different CdCl_2_ Conc. On tolerance index (TI) of *A. niger* and *A. terreus* On PDA medium

Cd conc. (mg/kg)	*A. niger*	*A. terreus*
	TI	TI
0	0	0
50	100	100
100	100	5.24
150	45.12	4.87
200	37.31	19.02
250	27.56	1.95
300	10.97	12.56
350	7.31	0
400	6.09	0
450	0	0

*The data in the table was mean ± standard error of the means (*N* = 3). Letters indicate statistical significance at *p* ≤ 0.05 level

**Table 6:** Effect of different cd Conc. On the dry weight (g/L) and TI of *A. niger* and *A. terreus* On PDB medium

Cd conc. (mg/kg)	A. niger		A. terreus	
	Dwt (g/L)	TI	Dwt (g/L)	TI
0	6.48 ± 0.171a	0	6.1 ± 0.161a	0
50	6.35 ± 0.168ab	97.99	5.9 ± 0.156a	96.72
100	5.99 ± 0.158b	92.43	5.13 ± 0.135b	84.09
150	5.9 ± 0.156bc	91.04	4.6 ± 0.121c	75.40
200	5.5 ± 0.145c	84.87	4.4 ± 0.116c	72.13
250	0d	0	0d	0

*The data in the table was mean ± standard error of the means (*N* = 3). Letters indicate statistical significance at *p* ≤ 0.05 level

It was found that the Cd concentration had an effect on the fungal biomass of *A. terreus* and *A. niger*. *A. terreus* treated with 50 mg/L Cd did not differ statistically from the control culture, a decrease in the biomass with 100 and 150 mg/L Cd 5.13 and 4.6 g/L were obtained, compared to the control with 6.1 g/L (Table 6). However, a noticeable decline was obtained in the biomass at 200 mg/L Cd, with 4.4 g/L being obtained. Cao et al. [66] revealed that the biomass decreased with increasing concentration of As (V) or Cd (II) in HM-resistant endophytic fungi. To determine whether *A. niger* can grow in the presence of Cd, it was found that the fungus can grow in the culture medium with a 50 mg/L Cd concentration, as Luna et al. [39] stated that it was found that *A. niger* can grow up to 300 mg/L.

**Fig. 4:**
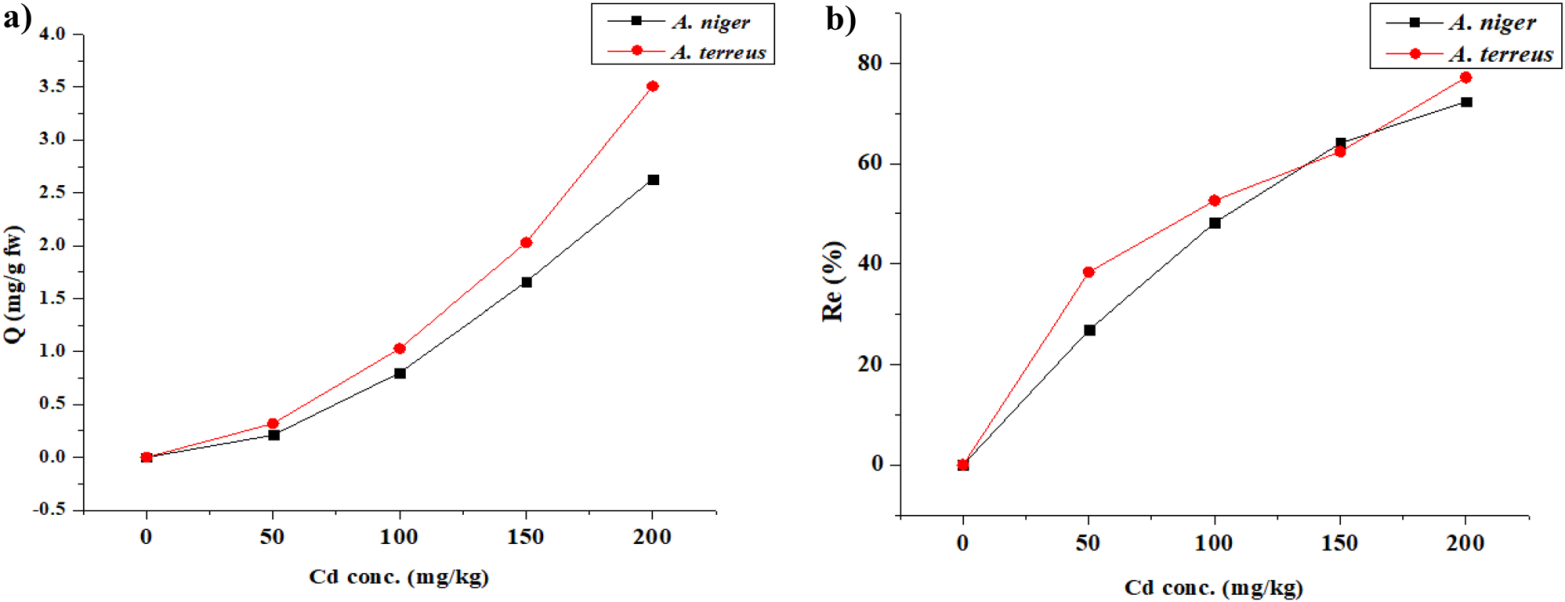
Effect of different Cd conc. on the bioaccumulation capacity (Q) and removal efficiency (Re%) of *A. niger* and *A. terreus* on PDB medium after 7 days

In the present study, the Cd tolerance of *A. niger* and *A. terreus* was up to 400 and 300 mg/L, respectively, and the Cd adsorption of viable mycelium was 2.63 and 3.51 mg/g at 200 mg/L, which exceeds the maximum permissible limit of the World Health Organization of 0.003 mg/L for Cd in drinking water [67]. Many workers stated that fungi possess a variety of mechanisms for metal stress [68, 69]. One of these strategies utilized by fungi is the binding of metal ions to functional groups, complexation with microbial extracellular polymer, and metal accumulation within the cells [70]. Also, according to Thatoi et al. [71], valence transformation, extracellular and intracellular precipitations, and active absorption are involved in the fungal detoxification of HMs in polluted environments. In our study, the living *A. niger* and *A. terreus* mycelium with high Cd adsorption capacity makes it a potential biological agent to remove Cd^2+^ from the contaminated environment. The removal rates from the medium were 48.20% and 52.63% with 100 mg/L Cd^2+^ concentration (Fig. 4). Our results confirmed that *A. niger* and *A. terreus* can remove Cd contamination.

### Cd removal efficiency by *A. niger* and *A. terreus*

The non-biodegradability of Cd and its considerable potential to cause negative impacts on microorganisms, ecosystems, and associated systems make it a serious worry for the developing world. HMs generate ROS that influences DNA formation, protein synthesis, and enzymatic functions [72], also binding to the surface of cells, leading to an imbalance in ions [73]. Fungi use two-step processes called biosorption and bioaccumulation to remove HMs (such as Cd^2+^) from soil and water [11, 67, 70]. In this study, we evaluated *A. niger* and *A. terreus* with remarkable Cd uptake and removal capacity. The results proved that *A. niger* and *A. terreus* were able to remove the Cd from the media. Our results agreed with Acosta-Rodríguez et al. [74], who stated that multiple metal-resistant *A. niger* were able to eliminate different metal ions up to certain levels. Additionally, the removal percentage depended on the initial concentration used. Also, our results in Tables (4 and 5) illustrated that the *A. niger* exhibited higher growth in Cd^2+^-containing media in comparison to *A. terreus.* The percentage of removal of Cd was increased in *A. niger* with the increasing concentration of Cd from 50 mg/L to 200 mg/L (26.84–72.35%), indicating the relationship of metal uptake with biomass and dose of Cd (Fig. 4). However, it was found that uptake and % removal of Cd (II) from the liquid medium by *A. fumigatus* decreased with increasing concentration from 100 to 500 mg/L [75]. Our results also stated that at 200 mg/L Cd, the biomass of both fungi was decreased sharply and the percentage of removal was increased. Moreover, *A. terreus* fungal cells exposed to 150 and 200 mg/L of Cd showed higher metal uptake (Q) compared to cultures treated with 50 mg/L. The corresponding percentages of Re were 38.34, 52.63, 62.39, and 77.20% for cultures exposed to 50, 100, 150, and 200 mg/L of Cd, respectively (Fig. 4). Dijksterhuis and Wösten [76] stated that because it can produce a wide variety of metabolites, *Aspergillus* fungi are thought to have one of the most promising applications in biotechnology and industry. Additionally, *A. niger* has been extensively tested to be effective in leaching and remediating HMs, according to their capacity for physiological adaptation and tolerance resistance [39, 77].

### Physiological response of *A. niger* and *A. terreus* to cd stress

**Fig. 5:**
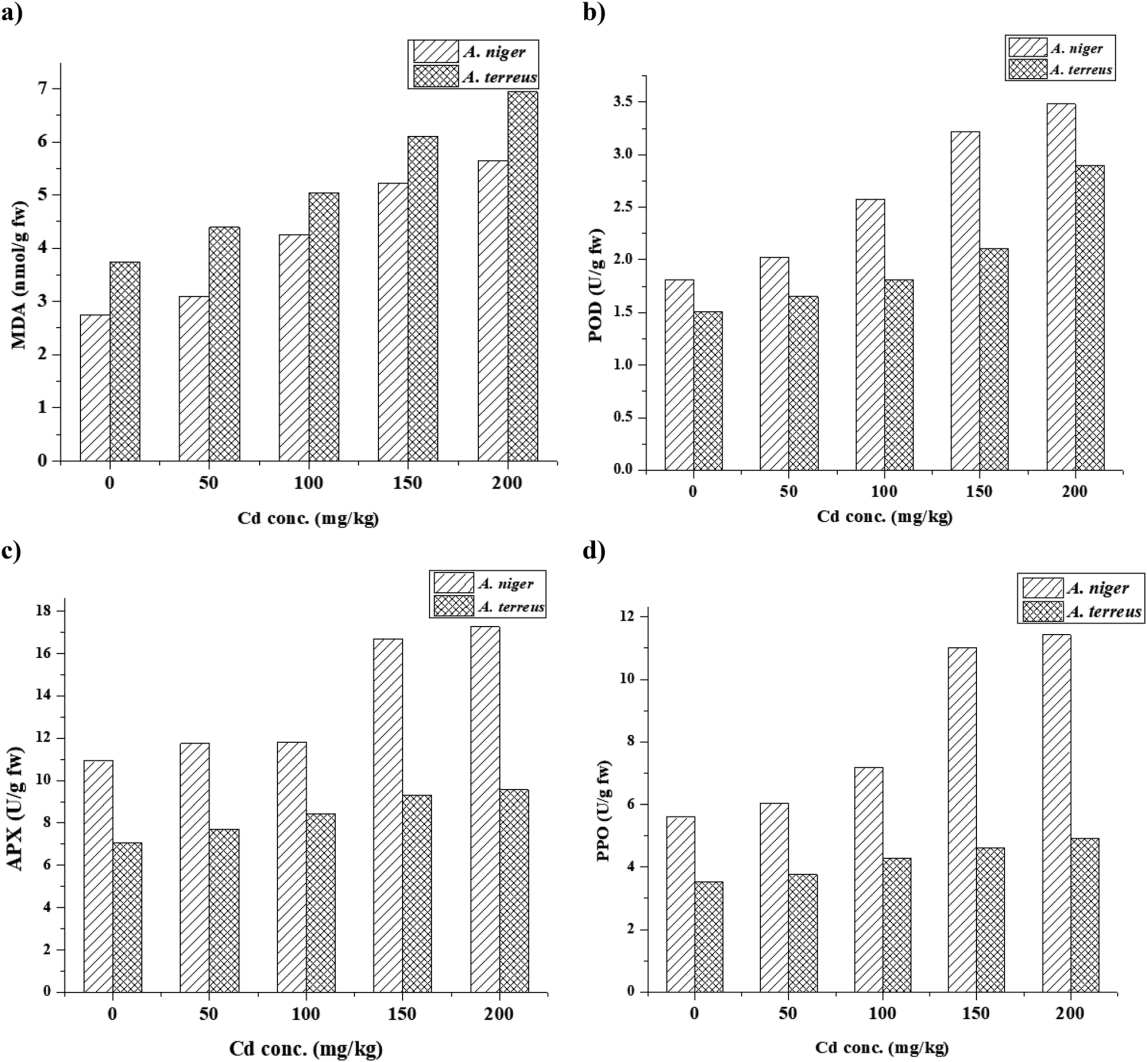
Effect of different Cd conc. on MDA and enzymatic antioxidants (POD, APX and PPO) enzymes activities of *A. niger* and *A. terreus* after 7 days

The oxidative stress induced by excess Cd generates ROS that react with the methylene groups of the unsaturated fatty acids of the plasma membrane, causing lipid peroxidation [28, 78, 79]. The primary metric for assessing membrane integrity is MDA, which is the byproduct of membrane lipid peroxidation [80]. The results obtained from the analysis of TBARS in response to the presence of Cd in the cultivation media are shown in Fig. 5a. MDA content was increased gradually by 12.95–105.95% (*A. niger*) and 17.27–85.38% (*A. terreus*), respectively, from 50 to 200 mg/L Cd treatments. Zhan et al. [81]. reported that Cd stress appreciably prompted the production of H_2_O_2_ and MDA in the mycelia of *Exophiala pisciphila*. These current findings show that oxidative stress resulting in membrane peroxidation is involved in the mechanisms of Cd toxicity. The fungal cell wall serves as their initial defense against Cd stress, and changes to the cell wall will unavoidably result in changes to the cell membrane [82]. A certain amount of Cd can be chelated by the cell wall to prevent damage to the membrane. Still, high Cd stress that surpasses the cell wall’s resistance threshold value will cause plasma membranes to peroxide, which will compromise the integrity and permeability of the membrane [83]. Besides, Paraszkiewicz et al. [84] listed that the mycelia of *Curvularia lunata* exposed to Ni^2+^ showed an increase in the TBARS levels over the control.

### PPO, POD and APX activities

ROS generated in fungal cells exposed to Cd stress disrupts cell organelles and interferes with several metabolic processes that regulate normal cell activity [28]. Thus, to withstand oxidative stress brought on by Cd, fungi have developed an antioxidant system in which antioxidant enzymes such as PPO, POD, and APX, and others are essential [85] in the microbial cells and are involved in the detoxification of ROS-generated stress [4]. The results presented in Fig. 5b, c, and d reveal the activity of antioxidant enzymes (PPO, APX, and POD) of *A. niger* and *A. terreus* were increased in response to Cd concentration (0, 50, 100, 150, and 200 mg/L). The same results were obtained by Todorova et al. [86], who found that the tolerance of *A. niger* to Cd (II) was correlated with the HM uptake, reactive oxygen species generation in the cells, and the efficiency of the antioxidative defense system. Compared to the non-stressed *A. niger* (Cd-free), the POD and PPO activities were enhanced by 92.00 and 104.24% at 200 mg/L Cd. APX activity was increased by 58.12% at 200 mg/L. The activities of antioxidant enzymes by *A. niger* and *A. terreus* were increased and subsequently responsive to Cd stress till their maximum values. It is observed that the Cd presence in the culture medium increased antioxidant activities compared to the control (*p* < 0.05) as a cellular response [87]. This result indicates an induction mechanism of ROS formation. The increase in enzyme activities is a mechanism for Cd detoxification via ROS degradation. *A. niger* and *A. terreus* had different protective mechanisms in response to Cu stress, as Luna et al. [39] reported. It was documented that HM stress causes an increase in antioxidant activity in a variety of fungal isolates [88]. Therefore, it can be inferred from the changes in MDA, mycelial growth, and morphology that antioxidant enzymes are essential for Cd detoxification and that *A. niger* has a more potent antioxidant system, which leads to its higher tolerance to Cd.

### Effect of cd stress on non-enzymatic antioxidants [total soluble protein (TSP), proline, and total soluble carbohydrates (TSC) contents] of *A. niger* and *A. terreus*

**Fig. 6:**
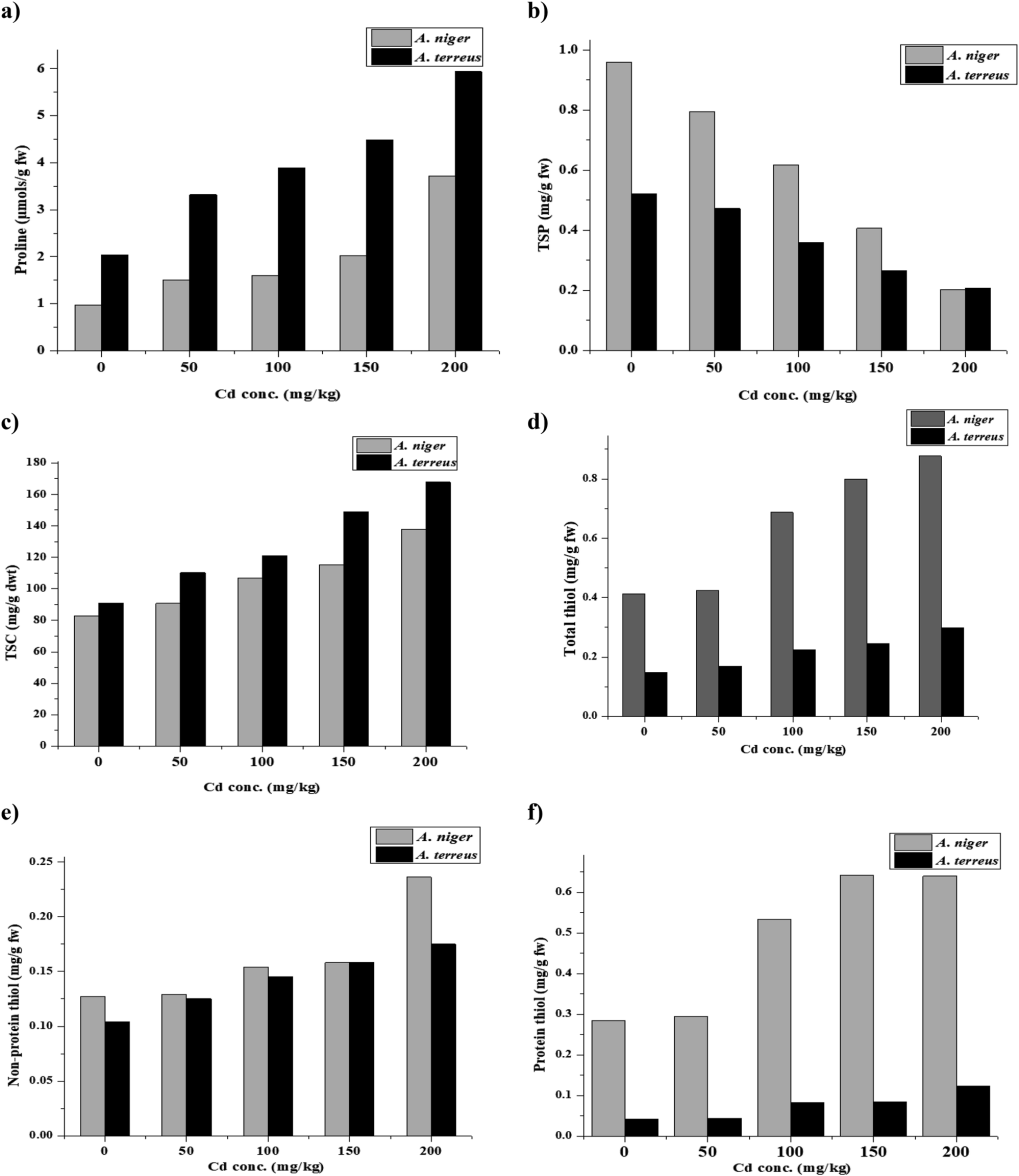
Effect of different Cd conc. on non-enzymatic antioxidants (proline, total soluble carbohydrates [TSC], total soluble protein [TSP]), total thiol, non-protein thiol and protein thiol of *A. niger* and *A. terreus*

Variations in the soluble protein profile in *A. niger* and *A. terreus* under different Cd concentrations were studied as appeared in Fig. 6. The trend of decreasing in the intracellular protein content of *A. niger* with different Cd concentrations was similar to that of *A. terreus*. A maximum decrease in proteins was observed with the highest Cd concentration in both fungal strains at 79.02 and 60.34%, respectively, compared to the control. Our results are divergent from El-Sayed and El-Sayed [28], who reported that proteins of *F. solani* were increased with increasing Ag concentration. However, 400 mg/L Ag(I) reduced total soluble protein contents, which could result from a high Ag(I) intolerance. Guelfi et al. [89] ascribed ascribed the decrease in *A. nidulans’* protein content to the mycelium’s autolysis and subsequent proteolytic disintegration in the presence of elevated Cd concentrations. Moreover, both free proline and TSC have been shown to accumulate in response to abiotic stress, and their accumulation may play a role in protecting the fungus against the adverse effects of stress. It has been found that proline serves as a marker for the main metabolic responses to stress. Moreover, it may detoxify free radicals, and their accumulation assists organisms in lessening oxidative stress [90–92]. In this study, an increased proline level was observed with increasing Cd concentration in both fungal isolates as 3.721 and 5.937 µmols/g fw; respectively, compared to the control (Fig. 6), indicating that an elevation in HM oxidative stress may result in a proline accumulation. This demonstrated that proline protects enzymes, preserving osmotic equilibrium and cell membranes. Our findings are consistent with Raj and Mohan [93] and Kumar and Dwivedi [94] that high levels of intracellular proline are specific characteristics of hypertolerant HMs and have a functional role in metal resistance. Regarding TSC, our results also demonstrated that TSC increased significantly in stressed fungi, 137.8 and 167 mg/g dwt, compared to the control one, especially at a high Cd concentration (200 mg/L). In harmony with this finding of Kanwal et al. [95], who stated the accumulation of TSC contents under different concentrations of HM stress. The excessive accumulation and production of many osmolytes is a critical defensive mechanism under metal stress [96].

### Effect of cd on thiol content of *A. niger* and *A. terreus*

Numerous techniques have been developed by fungi for dealing with exposure to detrimental HM concentrations. Among these techniques, the chelation in extracellular elements of the cell wall, intracellular detoxification, and vacuolar sequestration of HMs are the first line of defense that hinders the interaction of HMs with cell constituents [9, 97]. Furthermore, a variety of enzymatic and non-enzymatic antioxidant responses to lessen the impact of HMs within the cellular structures were reported. Thiols are essential components for HM tolerance to maintain the cells’ redox equilibrium [98]. Thiol contents by *A. niger* and *A. terreus* were gradually increased with the Cd stress during growth (Fig. 6). A strong increase in total thiol (0.299 and 0.877 mg/g fw) in *A. terreus* and *A. niger* was recorded at a concentration of 200 mg Cd/L as compared to their controls (0.147 and 0.412 mg/g fw). Thiol’s elevated levels upon metal exposure confirmed its significance for the survival of fungi. Members of the thiol family are capable of binding HM ions via thiolate coordination in fungi [88, 99].

### Role of organic acids in cd tolerance

**Fig. 7:**
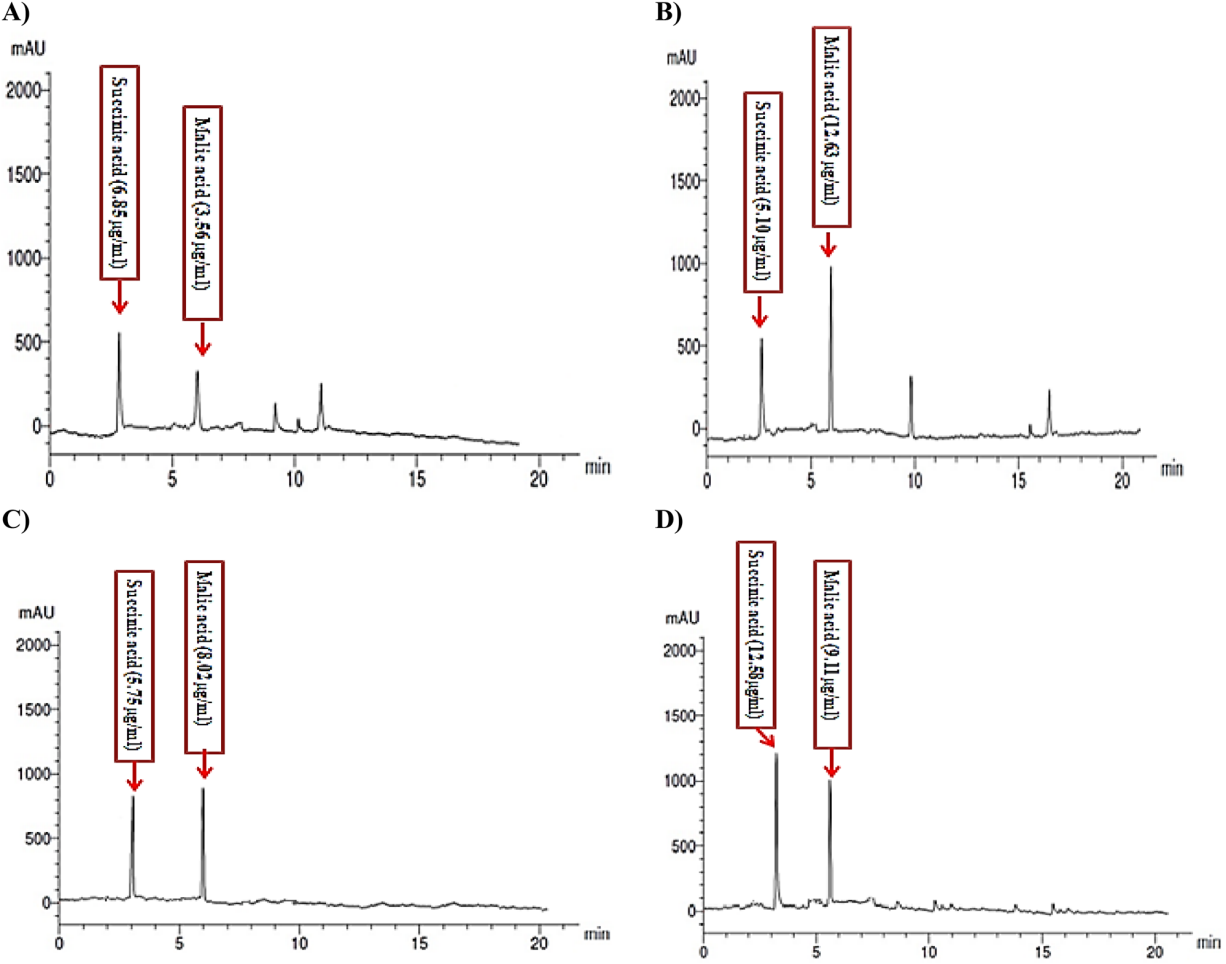
HPLC chromatograms of Cd-free culture filtrate (control) of *A. niger* and *A. terreus* (**A** and **C**; respectively)) and Cd-supplemented culture filtrate (200 mg/L) of *A. niger* and *A. terreus* (**B** and **D**; respectively)

Low molecular weight organic acids (LMWOAS) produced by fungi, such as citric, oxalic, and gluconic acids, have been established to be vital for the removal and solubilization of some HMs [9, 100]. Through their extracellular and intracellular interactions, these organic acids help fungi adapt to and tolerate high concentrations of HMs [101]. Figure (7) showed very clear differences in the malic and succinic acid production in the presence or absence of Cd in *A. niger* and *A. terreus*. The most remarkable difference was the production of malic acid in the presence of Cd in *A. niger* and succinic acid in *A. terreus* (Fig. 7). Malic acid in *A. niger* was increased around four-fold in the presence of Cd, while succinic acid showed a decrease in its concentration. *A. niger* was chosen because of its potential for biotechnological applications in the synthesis of enzymes and its ability to create organic acids, particularly citric acid [37]. Din et al. [102] have noticed a considerable increase of organic acids such as gluconic, oxalic, and fumaric acids by *A. tubingensis*, which resulted in Cd, Co, and Ni bioleaching with an efficiency of more than 50%. Priyanka and Dwivedi [9] stated that proteins, enzymatic and non-enzymatic antioxidants in addition to different organic acids are essential for HMs’ detoxification. HPLC chromatograms of Cd-free control and Cd-stressed *A. niger* (200 mg/L) showed that malic acid concentrations were 3.56 µg/mL and 12.63 µg/mL, respectively (Fig. 7a and b). Cd prompted the production of malic acid (254.77%, as compared to the control). In the presence of Cd, an increase in LMWOAS may have an impact on chelation, which could lessen the metal’s toxicity and increase its accumulation [85]. Under Cd stress, *Agaricus bisporus’s* LMWOAS was crucial for both detoxification and survival [103].

## Conclusion

It is found that there is an influence of Cd on both radial growth and dry biomass of both fungal species in this work, and a notable decline in biomass concentration of 200 mg/L is recorded. *A. niger* and *A. terreus* show a decrease in the soluble protein content directly related to the Cd concentration. Additionally, the MDA content is significantly increased as a result of Cd exposure. *A. niger* and *A. terreus* exposed to Cd exhibit an increase in the activities of PPO, APX, and POD, revealing the antioxidant response activation against metal stress. The maximum Cd removal rate of both fungal isolates is at 200 mg/L. Through these observations, we find that *A. niger* and *A. terreus* are powerful fungi that can withstand elevated Cd concentrations and are beneficial microbial organisms that successfully lower soil contamination from Cd and have the potential for bioremediation. These kinds of data are essential to the development of bioprocesses in industrial and environmental domains, as they help to clarify the cellular and molecular capabilities of these isolates.

## Data Availability

Sequence data that support the findings of this study have been deposited in the NCBI under accession numbers; PQ846493 and PQ846076.

## Abbreviations

APX: Ascorbate Peroxidase
kd: Growth Rate
TI: Tolerance Index
PPB: Potassium Phosphate Buffer
HPLC: High Performance Liquid Chromatography
HMs: Heavy Metals
POD: Peroxidase
PPO: Polyphenol Oxidase
MICs: Minimum Inhibitory Concentrations
MDA: Malondialdehyde
ROS: Reactive Oxygen Species
Re%: Removal Efficiency
Qss: Bioaccumulation Capacity
